# Plain set and stirred yogurt with different additives: implementation of food safety system as quality checkpoints

**DOI:** 10.7717/peerj.14648

**Published:** 2023-01-27

**Authors:** Aya Allam, Noha Shafik, Ahmed Zayed, Ibrahim Khalifa, Ibrahim A. Bakry, Mohamed A. Farag

**Affiliations:** 1 Chemistry Department, School of Sciences & Engineering, The American University in Cairo, Cairo, Egypt; 2 Pharmacognosy Department, College of Pharmacy, Tanta University, Tanta, Egypt; 3 Institute of Bioprocess Engineering, Technical University of Kaiserslautern, Kaiserslautern, Germany; 4 Food Technology Department, Faculty of Agriculture, Benha University, Benha, Egypt; 5 Food and Dairy Technology Department, Faculty of Technology and Development, Zagazig University, Zagazig, Egypt; 6 Pharmacognosy Department, Faculty of Pharmacy, Cairo University, Cairo, Egypt

**Keywords:** Yogurt, HACCP, Safety, Contaminations, Fermentation

## Abstract

Hazard Analysis Critical Control Point (HACCP) is a risk management protocol developed to ensure food safety through a precautionary approach that is believed to offer assurances in producing safe food for customers. Yogurt is made in a number of phases, commencing with the collection of raw milk and ending with consumer consumption. While this is happening, major economic and health issues might arise from exposing the manufacturing line to biological, chemical, and/or physical contaminations. As a result, the decision tree approach was used to determine the CCPs during the production of yogurt. Additionally, biological dangers are incorporated as a by-product of the system’s implementation performance. In particular, the plain set and nut puree-honey-fortified stirred yogurt manufacturing techniques are highlighted for the first time in this study. The potential manufacturing risks are described for the first time, together with information on how HACCP plans may guard against major risks that could result in the production of yogurt that is not in compliance with established standards.

## Introduction

Yogurt is one of the most well-known dairy products, well recognized for its health benefits. Yogurt presents an excellent source of vital components and probiotics. It is a food product that can be advised for patients with gastrointestinal troubles and lactose intolerance (Masoumi et al., 2021; Morelli et al., 2019). Nevertheless, it can present a potential source of foodborne pathogens (Berge & Baars, 2020). Such biological hazards can be introduced during yogurt manufacturing by using poor quality raw milk, improper chilling, and inadequate packaging, which can offset yogurt quality and safety attributes. To illustrate, fermented dairy products are typically associated with concerns about microbiological food safety and deterioration. Typical example of foodborne illnesses linked to dairy products include *Brucellosis*, *Salmonella, Listeria, and Clostridium botulinum* (Garcell et al., 2016; Ripolles-Avila et al., 2020). Milk and dairy products contribute to 2–6% of the foodborne illness outbreaks in developed nations (Claeys et al., 2013). Moreover, contamination caused by fungi such as yeast or mold poses a comparable problem than that caused by bacteria (Boor et al., 2017). In March of 2021, the US FDA reported that yogurt products were contaminated with *Mucor circinelloides*, a mucoralean fungal pathogen, and more than 200 consumers complained of symptoms, including vomiting, nausea, and diarrhea (Lee et al., 2014). Therefore, strict food safety measures are warranted for such a critical food industry.

Visual inspection of the finished product was previously the typical approach; however, such testing is not adequate to ensure complete safety of dairy products (Cusato et al., 2013). Currently, compliance with good manufacturing practices (GMP), sanitation standard operating procedures (SSOP), good hygiene good practices (GHP), sometimes known as operational requirements programs (OPRPs), and HACCP principles are now required for safe food production (Kamboj et al., 2020). HACCP system’s major goal is to be preventive in nature by analyzing biological, chemical, and physical dangers that influence the entire food production chain (Allata, Valero & Benhadja, 2017; Manley, 2011). Several studies (Chen et al., 2019; El-Hofi, El-Tanboly & Ismail, 2010; Manley, 2011) reported that using HACCP plans improved the microbiological quality of food items. HACCP is thus established on predicting hazards and controlling measures throughout the production process rather than relying on testing the final product (Pardo et al., 2013). Despite that, implementing HACCP for fermented milk production faces significant challenges including a lack of knowledge, motivation, and confidence in food safety legislation, as well as a lack of financial resources and human resource limitations (Amagliani et al., 2012; Jianu & Chiş, 2012; Yapp & Fairman, 2006). Moreover, if the manufacturing process changes for any reason, the HACCP plan must be modified as well.

This review focuses on identifying the critical control points identified in yogurt production of different types, and analysis of hazards and limitations to be implemented for establishing an efficient HACCP system. An account of yogurt’s production, classification, HACCP plans, and the hazard assessment for every material and step to be used will be presented. Most importantly, critical control points for the plain set yogurt, stirred yogurt with fruits, nuts, and honey are presented highlighting specific CCPs for each type.

## Materials and Methods

Literature was reviewed using electronic databases, including Web of Science, Science Direct, PubMed, Google Scholar, Wiley, and SciELO. This review included English language publications published in peer-reviewed journals between the earliest record date till June 28^th^, 2022. The search results were obtained by utilizing phrases such as “Plain set and stirred yogurt” OR “HACCP”; “Food safety,” OR “Check points.” Duplicate publications were eliminated manually, yielding a total of 80 peer-reviewed articles. Articles for which no full access was found were excluded such as conference proceedings or articles not published in English.

### Approach

The implementation of the food safety system incorporates several procedures, for instance, GMP, SSOP (Standard Operation Procedures), and HACCP, alongside the implementation of TQM (Total Quality Management) systems *i.e*., 9,000-series. The success of HACCP requires management commitment to ensure full adherence to these practices (Codex Alimentarius, 2020). Besides, HACCP is a mandatory requirement for all EU members by the 92/46 directive (Dairy Hygiene) (Chountalas, Tsarouchas & Lagodimos, 2009). To implement HACCP plans, all prerequisite programs should be adopted and maintained. Prerequisite programs (PRP) include: GMP, SSOP, QA (quality audit), QC (quality control) preventive maintenance, sanitation program, and microbiological analysis (Park, 2010). The main goal of this study was to determine the application and efficacy of HACCP for plain set and stirred yogurt with different additives to guarantee the production of a safe product.

### Procedure

HACCP system is based on the following seven principles that need to be well executed (Cusato et al., 2013; Merzlov et al., 2018; Sandrou & Arvanitoyannis, 2000) including: (I) Hazard analysis: to allocate the potential hazardous factors in every processing step in yogurt production, raw materials, and processing environment, such as microbiological (*i.e*., bacteria, viruses, coliforms), physical (*i.e*., metal, glass, plastic, wood, *etc*.), and chemical (drug residues, mycotoxins, and antibiotics); (II) Identification of CCPs: to control and prevent any of the previously mentioned hazards specifically if they are likely to occur; (III) Critical limits: to define acceptable range of any factor in the CCP; (IV) Monitoring needs: to include lab tests, assigned personnel, visual inspection, and frequency of monitoring; (V) Corrective actions: to be taken if there is any detected variation during monitoring; (VI) Keeping records: any records kept throughout monitoring phase or corrective action including recording frequency of cleaning storage tanks or records of pasteurization temperature; (VII) Verification: procedures done to confirm the effectiveness of the developed HACCP plan.

Tables 1–5 are used to display all elements that comprise the seven principles of HACCP, including:

1. The laboratory steps applied through the production process highlight corrective actions.
2. The type of hazards and control measures for raw milk and non-dairy ingredients highlighting corrective actions.
3. The control measures for storage and pasteurization stages highlight corrective actions.
4. The control measures for fermentation and packaging stages highlight corrective actions.
5. The control measures for fruit puree, nut puree, and honey highlighting corrective actions.

**Table 1:** The laboratory steps applied through the production process highlighting corrective actions.

Materials	Processing steps	Microbiological tests	Chemical tests	References
Raw milk	Receiving	Total count and spore count.	Temperature, pH, and fat (%).	Chandan et al. (2006) and Mostert & Jooste (2005)
Pasteurized milk	Pasteurization	Alkaline phosphatase (ALP), and laboratory pasteurization count (LPC).	Temperature	Chandan et al. (2006) and Payne & Wilbey (2009)
Standardized milk	Mix and preparation.	Total count, coliform count, and yeast and mold count.	Fat % and total solids.	Chandan et al. (2006)
Starter		Gas bubbles.	Lactic acid and the coagulation time.	Tamime & Robinson (2007)
Contact surfaces and tanks	All steps	Swab rinse method	—	Mostert & Jooste (2005)
Packages	Packaging	—	Metal detector & automatic photoelectric cell.	Chountalas, Tsarouchas & Lagodimos, (2009)
Storage areas	Storing	—	Temperature and humidity.	Sandrou & Arvanitoyannis (2000)

**Table 2:** The type of hazards and control measures for raw milk and non-dairy ingredients highlighting corrective actions.

Hazard	Type of hazard	Control measure or corrective action
		– Ensure that transporting trucks are regularly sanitized (with records) and that the required storing temperature has been maintained during transport (Food & Drug Administration, 2018).
Biological	Presence of vegetative bacteria.	– The unloading equipment and area must be kept clean with maintaining adequate separation from surroundings (Food & Drug Administration, 2018).
		– Supplier must provide proof of analysis such as certificate
	Presence of antibiotics or veterinary drugs.	- Test all containers and tankers for any residues of animal medications (Food & Drug Administration, 2018).
Chemical	Presence of mycotoxins.	– Supplier must provide letter of guarantee due to possible risk of fungal contamination of raw milk from animal feed, aflatoxin can be present in raw milk (Food & Drug Administration, 2018).
		– The transporting vehicles must be inspected during receiving for any possible risk that may cause cross contamination to the product.
	Remains of sanitizing chemicals.	– Ensure adequate separation between the vessels and lines used for the product and lines used for detergents and cleaning chemicals (Food & Drug Administration, 2018).
		– Any surface that comes in contact with the product should be sanitized before using every time (Poltronieri & Rossi, 2017).
Physical	Presence of foreign substances.	– Obtain raw milk from grade “A” IMS listed sources (Food & Drug Administration, 2018).
	Contamination with foreign materials	The transporting vehicles must be inspected during receiving for any possible risk that may cause cross contamination to the product

**Table 3:** The control measures for storage and pasteurization stages highlighting corrective actions.

Hazard	Type of hazard	Control measure or corrective actions
		– Raw milk must be stored at temperature not more than 10 °C.
		– Milk must be adequately heated (for batch pasteurization: 63 °C for 30 min).
	Vegetative bacteria growth.	– Keep all lines, vessels and valves clean and sanitized with records of the time and date according to recommended frequency (Food & Drug Administration, 2018).
Biological		– The product shall be considered to be disposed if the temperature or time are not met within 2 h (Food & Drug Administration, 2018).
		– Pasteurizer design and construction must meet the Grade-A Pasteurized Milk Ordinance (Poltronieri & Rossi, 2017).
		– Storage vessels must be cleaned after each use (keep records).
	Contamination with vegetative bacteria.	– Linked valves and lines must be cleaned at least once daily after using them.
		– Connected opening and valves must be tightly closed with proper lids. The product-contact surfaces of all multi-use containers, utensils and equipment used in storage shall be effectively cleaned and shall be sanitized before each use (Food & Drug Administration, 2018).
		– Ensure adequate separation between the vessels and lines used for the product and lines used for detergents and chemicals (Food & Drug Administration, 2018).
	Remaining cleaning and sanitizing chemicals.	– To ensure that needed separation is maintained, inter-washes throughout the day can be done (Food & Drug Administration, 2018).
Chemical		– Any surface that comes in contact with the product should be sanitized before using every time during processing (examples of contacts surfaces: equipment and tanks used for different purposes) (Poltronieri & Rossi, 2017)
	Contamination with allergens from materials mixed with product.	– Pipelines, valves, and any part of the pasteurizer used for any other food, that might contain allergens, must be cleaned properly to avoid unlabeled cross contamination (Food & Drug Administration, 2018).

**Table 4:** The control measures for fermentation and packaging stages highlighting corrective actions.

Hazard	Type of hazard	Control measure or corrective actions
		– Appropriate and safe bacterial cultures can be added directly without pasteurization.
		– Any opening in the vat must be closed tightly and all surrounding conditions must be controlled.
		– In case of open vats, individual rooms are required (Food & Drug Administration, 2018).
Biological	Contamination with vegetative bacteria.	– Packaging materials must be provided by suppliers on Interstate Milk Shippers (IMS) list with letter of guarantee.
		– Supplier should provide certificate of analysis.
		– Single service packages when received must be protected from contamination.
		– Any equipment used in packaging must be easily cleaned and regularly inspected (Food & Drug Administration, 2018).
		– For single-use containers as those used in yogurt production to be acceptable: Coliform bacteria are not detected. Testing for residual bacterial count is done on four random samples taken on the same day and, in three of them, the residual bacterial count is 50 or less colonies per container (Poltronieri & Rossi, 2017).
	Toxin production.	– Slow vats are required to avoid risk of toxins in the final product (Food & Drug Administration, 2018).
Chemical	Remaining cleaning and sanitizing chemicals.	– Ensure adequate separation between the vessels and lines used for the product and lines used for detergents and chemicals and prevent any cracks to avoid leakage (regular maintenance required) (Food & Drug Administration, 2018).
		– Any surface that comes in contact with the product should be sanitized before using every time during processing (examples of contacts surfaces: equipment and tanks used for different purposes) (Poltronieri & Rossi, 2017).
	Contamination with carcinogenic chemicals.	– Supplier must provide letter of guarantee.
		– Purchase from suppliers on IMS list.
	Food allergens contaminating products not labeled to be allergenic.	– Any packaging equipment used for yogurt and any other food containing allergens must be cleaned properly to prevent cross contamination (Food & Drug Administration, 2018).
		– All inlets in cultured product vessel must be tightly controlled.
Physical	Foreign substances.	– The associated equipment must be maintained in good status to eliminate as source of hazard (Food & Drug Administration, 2018).
		– Ensure that the packaging zone is devoid of any glass pieces.

**Table 5:** The control measures for fruit puree, nut puree, and honey highlighting corrective actions.

Hazard	Type of hazard	Control measure or corrective actions
Biological	Contamination with vegetative bacteria.	– Adding fresh fruits with pH of ≤4.7, properly roasted nuts, and/or additives with water activity ≤0.85 to the dairy product after pasteurization does not affect the quality of the yogurt produced (Poltronieri & Rossi, 2017).
Chemical	Any chemical contaminants.	– Supplier should provide letter of guarantee for any additive added to the dairy product after pasteurization.
	Contamination with allergens from materials mixed with product.	– Pipelines, valves, and any part of the pasteurizer used for any other food, that might contain allergens, must be cleaned properly to avoid unlabeled cross contamination.
		– All inlets to cultured product tanks must be tightly controlled and closed.
Physical	Foreign substances	– Equipment must be maintained in good status to eliminate any source of hazard (Food & Drug Administration, 2018).

## Results and discussion

From literature, it is accepted that each dairy plant may adapt the flow diagram of yogurt processing for the HACCP plan depending on their own quality control system. Considering that there has not been such a comprehensive compilation of studies on this topic, we investigated pertinent related literature dating back as far as the late 1980s, however we primarily concentrated on publications from the last 10 years. Fermented dairy products still face several difficulties, including the need to improve science-based dairy safety strategies and the management of post-processing contamination, particularly on pathogens and causative organisms, despite significant advancements in reducing microbial safety and risk issues. Better methods must therefore be implemented and put into action for locating and identifying sources of foodborne infections. Additionally, alternative strategies must be developed to stop the spread of pathogens in the food production chain.

## Describe and identify product use

### Yogurt’s production

Dairy products are one of the major food industries that includes several products, including pasteurized milk, yogurt, cheese, butter, and others (Gao et al., 2021). In this report, we focus on yogurt’s production process and propose HACCP plan to improve its food safety. Yogurt is widely produced in the Middle East, Asia, Africa, and Europe (Surono & Hosono, 2011), with different products available in all food retailers and consumed by people of all ages (Chountalas, Tsarouchas & Lagodimos, 2009). Yogurt is considered the most popular fermented milk product due to its long shelf life and numerous health benefits (Boynton & Novakovic, 2014). It is a typical fermented dairy product produced using a starter culture to coagulate milk as the first step from cows, buffalos, goats, or sheep (Nagaoka, 2019). The finished products are typically packaged in plastic and stored in a refrigerator (0–6 °C) until their expiry date (mostly 14 d) (Chountalas, Tsarouchas & Lagodimos, 2009).

Heat-treated milk, including pasteurized and UHT milk are also used to produce a semi-solid yogurt with a custard-like texture. Starter cultures used to asset the fermentation process is a typical blend of *Lactobacillus delbrueckii* subsp. *Bulgaricus*, *L. acidophilus L. casei, Streptococcus thermophilus*, and *Bifidobacterium* spp. (Nagaoka, 2019). The fat content in the final product varies from 3.2% for full fat yogurt to 0.5% for non-fat yogurt, with 0.5–2% fat level in low-fat yogurt (Code of Federal Regulations (CFR) (2011) Title 21, Part 131). Accordingly, non-fat dry milk or condensed skimmed milk are used to increase the ratio of solids non-fat (SNF) (Sfakianakis & Tzia, 2014). Concerning nutrition, yogurt is a good source of essential minerals such as Ca, P, Mg, and Zn. Besides, Mg and Ca are available in ionic forms that increase their bioavailability and health benefits (Chandan & Nauth, 2012; Marette, Picard-Deland & Fernandez, 2017). In contrast, yogurt’s quality depends on numerous factors such as the manufacturing procedure, raw materials used, and the processing equipment. The two main types of yogurts present in the market include “set yogurt” which is more in a gel form, and “stirred yogurt” upon which the primary demand is due to its semi-liquid texture and sweet taste. The main difference among them during production lies in the fact that set yogurt is packed, then incubated, whereas stirred yogurt is incubated in tanks (Pagiataki, Chouliara & Kontominas, 2013). Other ingredients added during yogurt production to extend its flavor include sweeteners (sucrose, molasses, and high fructose corn syrup), flavoring ingredients, and color additives. Other components may be added for nutritional purposes, such as vitamins A and D (Jung, Lamsal & Clark, 2014) or for flavor enhancement such as fruit, juice, or others (Codex, 2003; Fisberg & Machado, 2015).

### Yogurt’s classification

**Figure 1:**
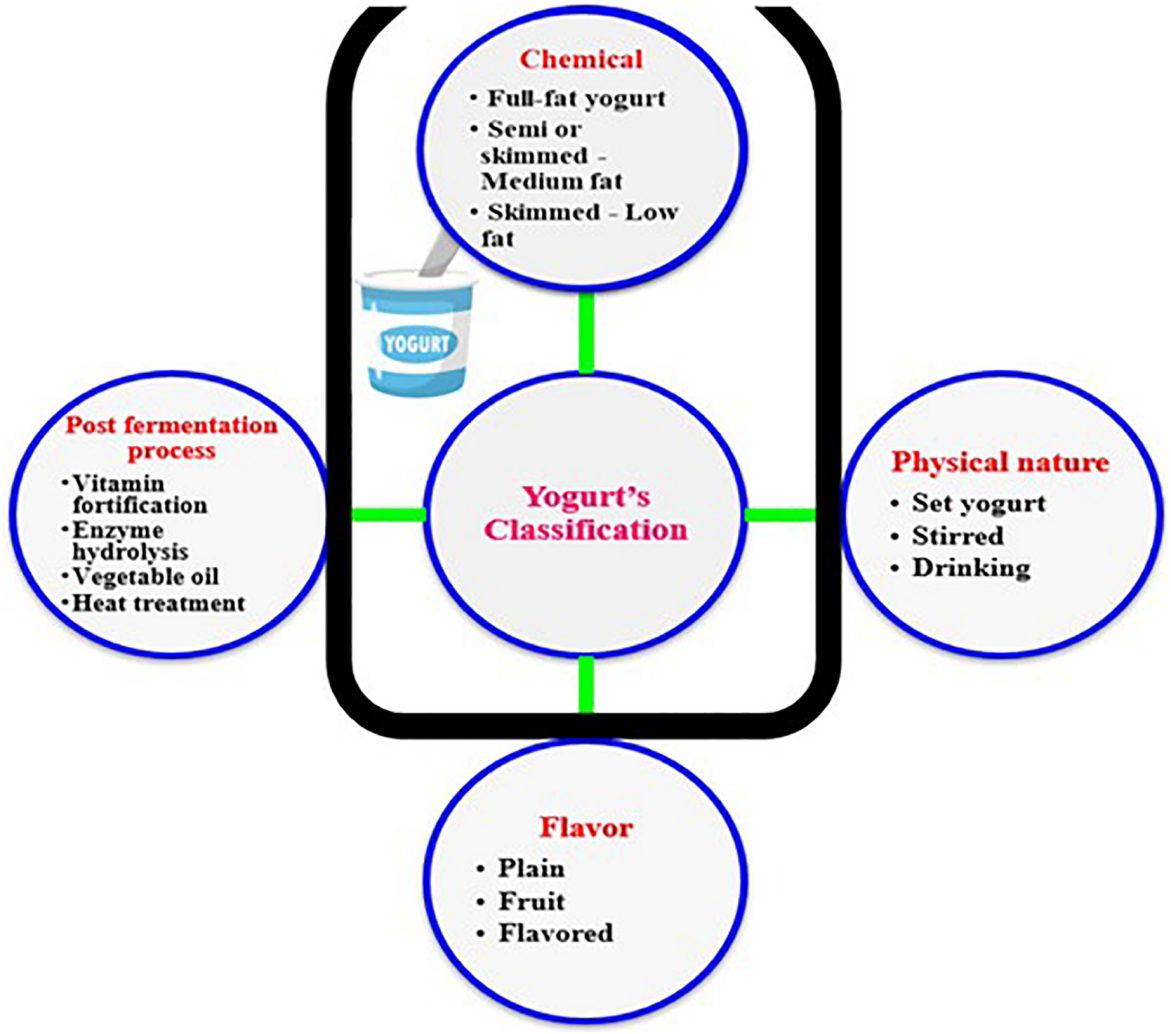
Diversity of yogurt products.

Various types of yogurts are typically manufactured to meet the wide variety of consumer preferences worldwide. As depicted in Fig. 1, yogurt products can be classified regarding production method, taste, and texture to the following types: (I) set yogurt which is incubated and cooled in final packages, with a jelly-like texture; (II) stirred yogurt which is incubated in tanks then broken to final coagulum prior to cooling, with a thick cream texture; (III) drinking yogurt which is incubated in tanks then broken final coagulum before cooling, with a liquid texture; (IV) frozen yogurt incubated in tanks then left to freeze, with an ice cream texture; (V) flavored yogurt which contains a high percentage of sugars and flavors with a structure similar to stirred one; (VI) concentrated yogurt in which water is removed with a gritty texture. Among these six types of yogurts, the set and stirred ones are the most common types of yogurts consumed worldwide, and therefore the main focus of this study.

### The production process and HACCP plans

**Figure 2:**
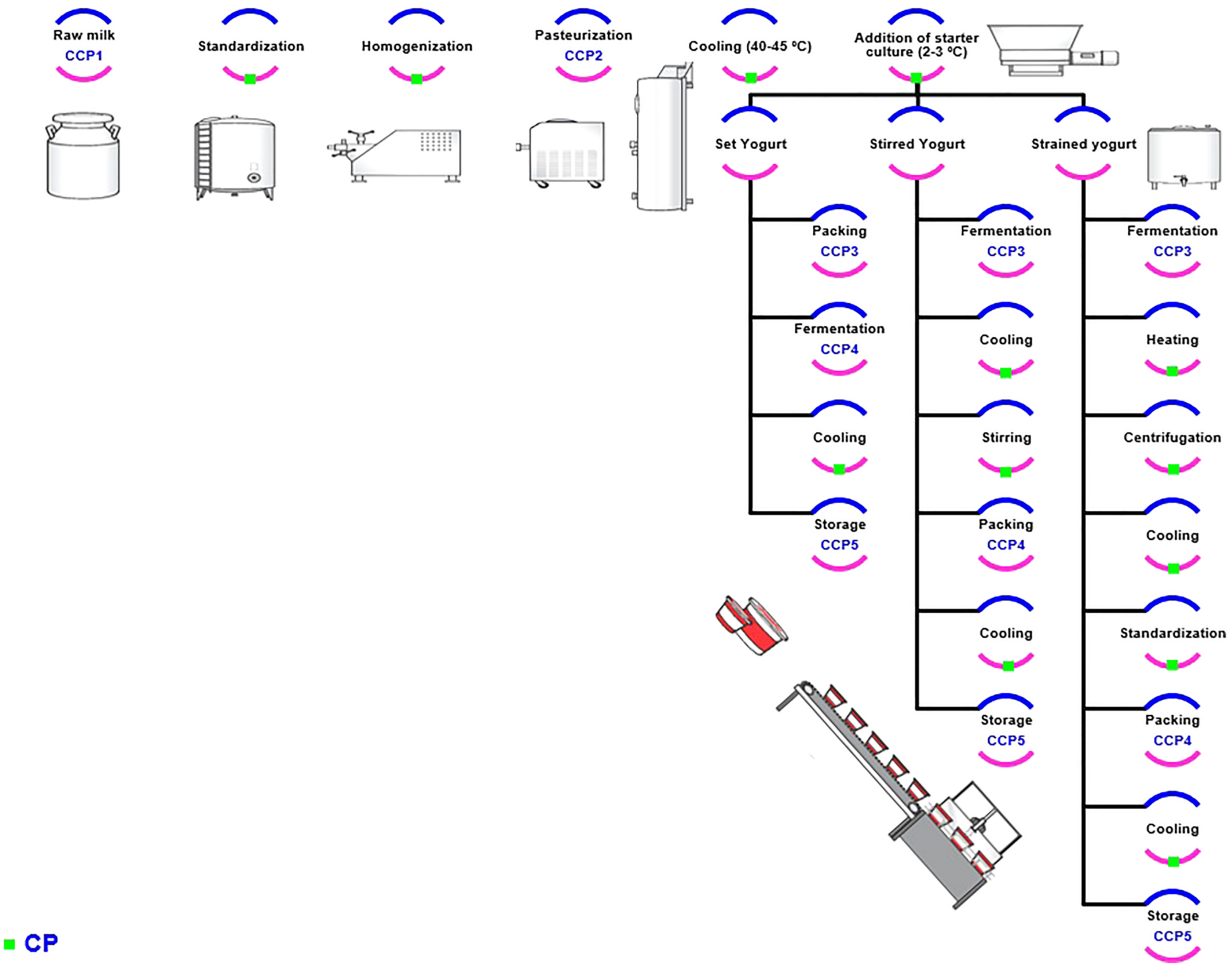
The flow chart of yogurt production (set and stirred & strained yogurt). The green arc indicated to the CP, where blue and pink arcs without green one referred to the CCPs, where the HACCP were planned for the whole process.

Two types of production processes are typically used to produce all yogurt types: the flow process and batch process. The flow process is used to create the product in a continuous process from raw material to the final product. On the other hand, in the batch process, production is divided into stages and tanks are used to store the semi-final material (Chountalas, Tsarouchas & Lagodimos, 2009). The various types of yogurts based on the different attributes are depicted in Fig. 1 (Griffiths, 2010). The flow chart used in planning HACCP for yogurt production is explained in Fig. 2. The raw materials used in yogurt production include raw milk, cream, starter culture, non-diary ingredients, and packaging materials, with each to hold certain hazard as explained (Table 1). The raw milk must be received and stored at 0–4 °C, filtered to remove foreign bodies, and centrifuged to a standardized fat and solid content (Arvanitoyannis & Mavropoulos, 2000). The cream also ought to be stored at 0–4 °C, whereas the starter culture is better to be stored at −40 °C. The non-dairy ingredients, which include fruit pulp, stabilizers, sweeteners, and coloring agents should also be stored in suitable conditions, with the perishable ingredients such as fruit purees being heated. On the packaging lines, packaging material must be sanitized prior to their filling with final products, and aseptic packaging should be utilized.

### Hazards assessment

HACCP aims at predicting and preventing microbiological, physical, and chemical hazards through assessing hazards of raw materials, processes, and final products. The objective of this stage is to collect and evaluate data on conditions of the whole process to identify potential hazards and eliminate them to an acceptable level. Herein, hazard assessment is divided into three main categories: raw materials, processing stages, and final products (Wallace et al., 2014) and discussed in the following subsections.

#### Hazard analysis of raw materials

##### Raw milk

Milk is the main component in yogurt production, with the quality of received milk imperative to control the quality of yogurt. The main risks associated with milk include biological hazards. Treatment of raw milk at the farm is a crucial stage, mostly using chilling to ensure eliminating microbial growth. Microbiological contamination influences the efficiency of pasteurization at a later stage, especially that some toxins produced by microorganisms are heat stable even under the low temperature which is commonly used to preserve the yogurt (>0–<10 °C). For instance, *Bacillus cereus* produces toxins at 6 °C; *Staphylococcus aureus* produces toxins at 10 °C, whereas *Salmonella typhi* at 15 °C (Table 2). Therefore, chilling milk in the farm is a mandatory step to ensure preventing any microbial growth, to be stored at 0–4 °C until proceeding with the processing step inside the dairy plant. Total bacterial count is an indicator of the farm conditions, where the raw milk should have a total bacterial count of <100,000 CFU mL^−1^, (Chountalas, Tsarouchas & Lagodimos, 2009). High somatic cell count is used to indicate and control the hygiene of livestock, which should be less than 4.0 × 10^5^ mL^−1^ (Arvanitoyannis & Mavropoulos, 2000).

Chemically, although antibiotics do not immediately impact consumer’s health, they inhibit the process of fermentation during yogurt production. Consequently, antibiotics testing is typically conducted in dairy factories to ensure their absence and the safety of milk. Organochlorine and organophosphorus are the two major pesticides affecting flavor alteration and/or starter growth inhibition (Tamime & Robinson, 2007). Organochlorine and organophosphorus residues in milk originate from contaminated feed, grass or corn silage, and direct application of pesticides on dairy cattle (Shaker & Elsharkawy, 2015). Lead, mycotoxin, and dioxins are the primary contaminants that may pass from the surrounding environment into the milk. Lead is associated with cardiovascular diseases and mental disorders, whereas mycotoxin and dioxins are carcinogens (Chountalas, Tsarouchas & Lagodimos, 2009). Milk contamination by heavy metals is a serious concern confronting the community’s health (El Sayed et al., 2011); the main source of contamination lies in grazing animals on contaminated fields (UNB, 2002).

Physically, any foreign fragments present in raw milk as straw, hair, or soil, are considered a high risk of contamination (Tamime & Robinson, 2007). In conclusion, received milk is expected to meet the following specifications including temperature of <10 °C, total count of <100,000 CFU mL^−1^, inhibitory substances of <0.007 IU mL^−1^, fat of ≥3.0 g, protein of ≥3.0 g, somatic cell count of <4.0 × 10^5^ mL^−1^, freezing point depression of <0.520 °C, and titratable acidity of <0.2% lactic acid (Tamime & Robinson, 2007).

##### Starter culture

Special emphasis is given to the quality of starter culture to assure safe conditions for yogurt fermentation, with yeast and molds for instance reported to influence the coagulation of yogurt and to further pose a health risk. Accordingly, it is essential to assess culture vitality by measuring the amount of lactic acid and coagulation time to be 0.6–0.7% and 3–4 h, respectively (Tamime & Robinson, 2007). The growth of yogurt bacteria is an essential process to control the quality of final products and manage the cost-effectiveness during manufacturing (Jung, Lamsal & Clark, 2014). To control fermentation and coagulation processes, starter culture must be free from foreign bacteria with excellent vitality (Chountalas, Tsarouchas & Lagodimos, 2009). Gas bubbles or nasty smells are considered indicators of infection or contamination by non-starter bacteria; the test of catalase reaction is conducted to confirm the presence of gross contamination in the starter culture (Tamime & Robinson, 2007).

##### Stabilizers

Stabilizers are used to maintain a reasonable degree of uniformity and to reduce wheying-off from the final product. A proper yogurt stabilizer forms gel structures in water, leaving less free water for syneresis action, and to increase yogurt shelf life. In addition, stabilizer should be effective at low pH without imparting any off flavor (Wang, Yuan & Yue, 2015). In the production of yogurt, modified starch, pectin, gelatin, and whey protein concentrates are the most used stabilizers; however, a combination of starch and gelatin is typical commercial mixture used in yogurt production. Whey protein is used due to their water-binding attributes (Jung, Lamsal & Clark, 2014).

##### Non-milk ingredients (fruit preparations, juice, sweeteners, flavor enhancer, and coloring agents)

Fruit-for-yogurt preparations are used to improve flavor and color; however, it must exhibit the identical taste and color of targeted fruit after mixing with yogurt (Table 2). Fruit preparation should also not lead to any phase separation or any defects in texture after blending with yogurt. It must have zero yeast and mold to assure the final product safety level (Jung, Lamsal & Clark, 2014). The microbiological hazards are associated with additives, specifically coliforms, yeast, and mold, which indicate a low level of hygiene (Temelli et al., 2006). Besides, fruits addition may represent a contaminated source with heavy metals and/or pesticide residues. Hence, all additives must comply with national and international legislation to alleviate hazards present above certain limits (Chountalas, Tsarouchas & Lagodimos, 2009).

#### Hazard analysis for processing stages

The reliable HACCP plan should consider the whole process of a product. Thus, we will subsequently discuss hazard analysis for each step of yogurt’s production in more detail in the next subsections.

##### Facility

It is imperative to control air quality inside the facility used as a process plant for yogurt production (Tamime & Robinson, 2007). Total count for bacteria, coliforms, yeast, and mold are valuable tests performed to evaluate the standard of plant hygiene, and to monitor the process of sanitation for all surfaces and dairy equipment (Mostert & Jooste, 2005).

##### Receiving

The chemical composition of raw milk is typically at 3.5% fat, 3.5% protein, 5% lactose, and 88% water (Chandan et al., 2006). Raw milk should only be received from good hygienic farms where animals are free from diseases and have not been treated with antibiotics (Sandrou & Arvanitoyannis, 2000). High coliform bacterial count is one of the approved tests that can reflect poor or unhygienic conditions of milking practices (Chandan et al., 2006). Some tests must be applied upon receiving the raw milk namely, temperature (10 °C), pH-value, antibiotic residues, somatic cell count (500,000–700,000 cells mL^−1^), bacterial limits (300,000 CFU mL^−1^ in the raw milk or 20,000 CFU mL^−1^ in the pasteurized milk), coliforms (not to exceed 10 CFU mL^−1^), and drugs (must be negative on drug residue) (Chandan et al., 2006). Mixing newly received milk with stored milk in the same tank should be strictly avoided to eliminate any contaminants; with all tanks to be cleaned and disinfected before storing received milk to eliminate the accumulation of milk residues. Inside the premises, milk must be conveyed in closed pipes throughout the whole production unit (Sandrou & Arvanitoyannis, 2000).

##### Mixture preparation and heat treatment

Standardization of milk fat, adding of stabilizers and sweeteners should precede heat treatment (Achaw & Danso-Boateng, 2021). Milk is typically treated at 85 °C for 30 min or 95–99 °C for 7–10 min to destroy all pathogenic microorganisms and most vegetative cells (Table 3). Heat treatment inactivates milk enzymes and produces suitable conditions to enhance the growth of the desired starter culture (Jung, Lamsal & Clark, 2014). Next, milk must be rapidly cooled to 4–5 °C (Sandrou & Arvanitoyannis, 2000). A variety of heat treatments can be applied to milk used for yogurt production including pasteurization (80–95 °C), sterilization (115–120 °C), and ultra-heat temperatures (135–140 °C) (Ritota et al., 2017). After ensuring that all raw materials are free from any hazards and safe to use, the hazard in this stage is limited to drop of temperature to 75 °C or below for at least 20 s (Chountalas, Tsarouchas & Lagodimos, 2009). Verifying the effectiveness of pasteurization, records of thermograph should be observed, and an alkaline phosphatase test (ALP) could be further employed to demonstrate the correct pasteurization of raw milk (Payne & Wilbey, 2009). Moreover, Laboratory Pasteurized Count (LPC) is a common test to confirm the absence of heat-stable bacteria as a high LPC will reduce shelf life of final products. For instance, *Bacillus cereus* is one of the microorganisms that can survive after pasteurization and increase LP. It should be noted that there are no approved limits for LPC by Federal Standards, but in some countries the maximum limit of LPC is 750 CFU mL^−1^ (Chandan et al., 2006).

##### Homogenization

At this stage, yogurt milk is an oil in water emulsion, and homogenization step is required to create a stable emulsion by reducing the size of the discontinuous phase. The objective of homogenizing milk is to reduce the diameter of fat globules to two microns, or less. The temperature must be around 50–65 °C and the pressure of 15–40 MPa (Dhankhar, 2014). Besides, the coagulations begin at pH 4.6–4.7, which is called the isoelectric point of casein. This step enhances texture development and eliminates wheying-off and surface creaming. Thus, sterilization of the homogenizer is considered an essential step to avoid contamination and must be operated under aseptic conditions (Papademas & Bintsis, 2010).

##### Fermentation

The fermentation room must be isolated from other areas of the plant to eliminate any cross-contamination and must be equipped with proper air ventilation. Fermentation tanks are insulated and wrapped in stainless steel with a cone bottom to drain any fluid after the incubation period, with temperature to be kept at 43 °C (Table 4). A clean-in-place (CIP) process is used to clean tanks with hot cleaning fluids (Jung, Lamsal & Clark, 2014). The starter could be a source of yeast and mold contamination; thus, it is imperative to enforce a strict examination of the starter to eliminate fungal contamination. A direct microscopic view of the used starter or plating of the starter on acidified potato dextrose agar is used to confirm the presence or absence of yeast and mold (Chandan et al., 2006).

##### Packaging and storage

Plastic containers are used to pack yogurt in various shapes, that characterize certain brands, and in different sizes, ranging from 113 to 904 g (Jung, Lamsal & Clark, 2014). The packaging material can present a source of microbiological contamination; hence, aseptic conditions must be applied at this stage. In addition, metal detectors are essential to detect and eliminate any physical hazards including foreign bodies or metal fragments (Chountalas, Tsarouchas & Lagodimos, 2009). Packages should be examined to prevent contamination to occur after filling or due to leakage (Sandrou & Arvanitoyannis, 2000). In case of using glass bottles, they must be inspected by an automatic photoelectric cell device to verify that all bottles are effectively cleaned and sanitized (Table 4). In addition, all suppliers of packaging materials should be audited to confirm their standards of bacteriological quality (Graves, Smith & Batchelor, 1998). All packaging materials must be stored in a room free of dust and humidity, with fumigation using iodine or chlorine regularly performed (Chandan et al., 2006).

The high temperature above 6 °C is the fundamental cause of spoilage during storage period, mainly by coliform, yeast, and mold (Viljoen et al., 2003). Hence, an automatic control loop should be used to alarm when the temperature rises above 6 °C in the storage areas (Sandrou & Arvanitoyannis, 2000). The fluctuating temperature during storage can increase fat oxidation and food spoilage. Besides, it can adversely affect the sensory properties and coagulum stability by changing texture and viscosity due to hydration of protein constituents and changing in the color of fruit as in case of fruit blended yogurt (Phosanam et al., 2021).

### Critical control points for production of plain set yogurt

The production of yogurt includes acid gelation of milk, which is typically generated by the fermentation of lactose to lactic acid by lactic acid bacteria. Different kinds of yogurts are produced by modifying processing conditions and the composition of the milk base. Set yogurt is incubated in retail containers to ensure that the gel structure remains intact. Whereas, stirred yogurt is incubated in large fermentation vessels and is agitated to obtain a smooth and viscous product before stuffing and packaging (Li, Ye & Singh, 2021).

#### Critical control points to produce plain set yogurt

**Figure 3:**
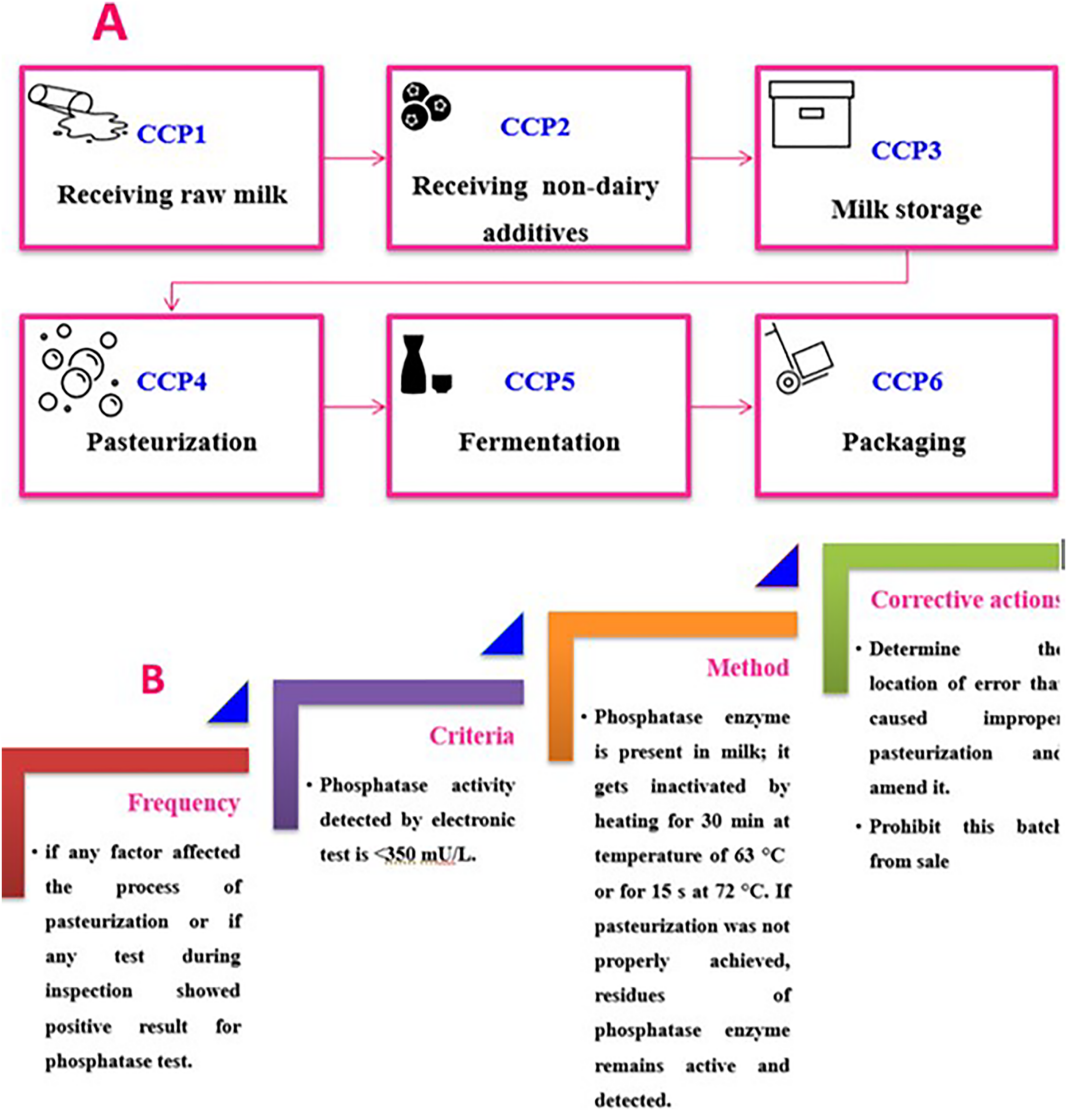
The main critical control points (A) in manufacturing of plain set yogurt (Merzlov et al., 2018), with some example for control measures applied for pasteurization point (B). Detection of the bacterial alkaline phosphatase for pasteurization efficiency.

Figure 2 shows the yogurt production scheme including the set and stirred one; the critical control points for yogurt product flow chart (CCP) would be discussed in detail in the following sections. Figure 3A shows the required CCPs, which will be discussed afterwards. There are six CCPs in the production of plain set yogurt starting from receiving the raw milk to reach the packaging step. Firstly, dairy products and raw milk should be stored ideally at a temperature of 7 °C or less. The control measures for raw milk and non-dairy ingredients are tabulated in Table 2. The center of the tank must also acquire the target storage temperature to ensure that the whole product is well stored as the middle of the container takes longest time to get cooled (Poltronieri & Rossi, 2017), as presented in Table 4. As milk represents a rich medium for the growth of microorganisms, pasteurization is used to destroy any possible pathogen present in milk. The International Dairy Federation defined pasteurization as, ‘‘A process applied to a product with the object of minimizing possible health hazards arising from pathogenic microorganisms associated with milk, by heat treatment, which is consistent with minimal chemical, physical and sensory changes in the product’’ (Papademas & Bintsis, 2010). It is well recognized that appropriate pasteurization is the sole applicable and commercially available procedure that can eliminate the risk of diseases arising from dairy products. However, efficiency of pasteurization is limited in eradicating risk of illnesses due to toxins produced by microorganisms in milk that could possibly exist owing to improper handling and/or storage. The effective pasteurization is achieved by maintaining the target heating temperature for specific time needed (time-temperature control). Consequently, the phosphatase activity test is used to determine the efficiency of the conducted pasteurization process (as shown in Fig. 3B) (Poltronieri & Rossi, 2017). Fermentation represents another key point that should also be critically controlled, especially the starter media and culture, as listed in Table 4. Packaging should be done at the same place as the final pasteurization step, and to minimize cross contamination (Table 4), with packaging equipment to have covers as a barrier between any contaminant and yogurt (Poltronieri & Rossi, 2017).

#### Critical control points of stirred yogurt with fruit puree, nut puree, and honey production

**Figure 4:**
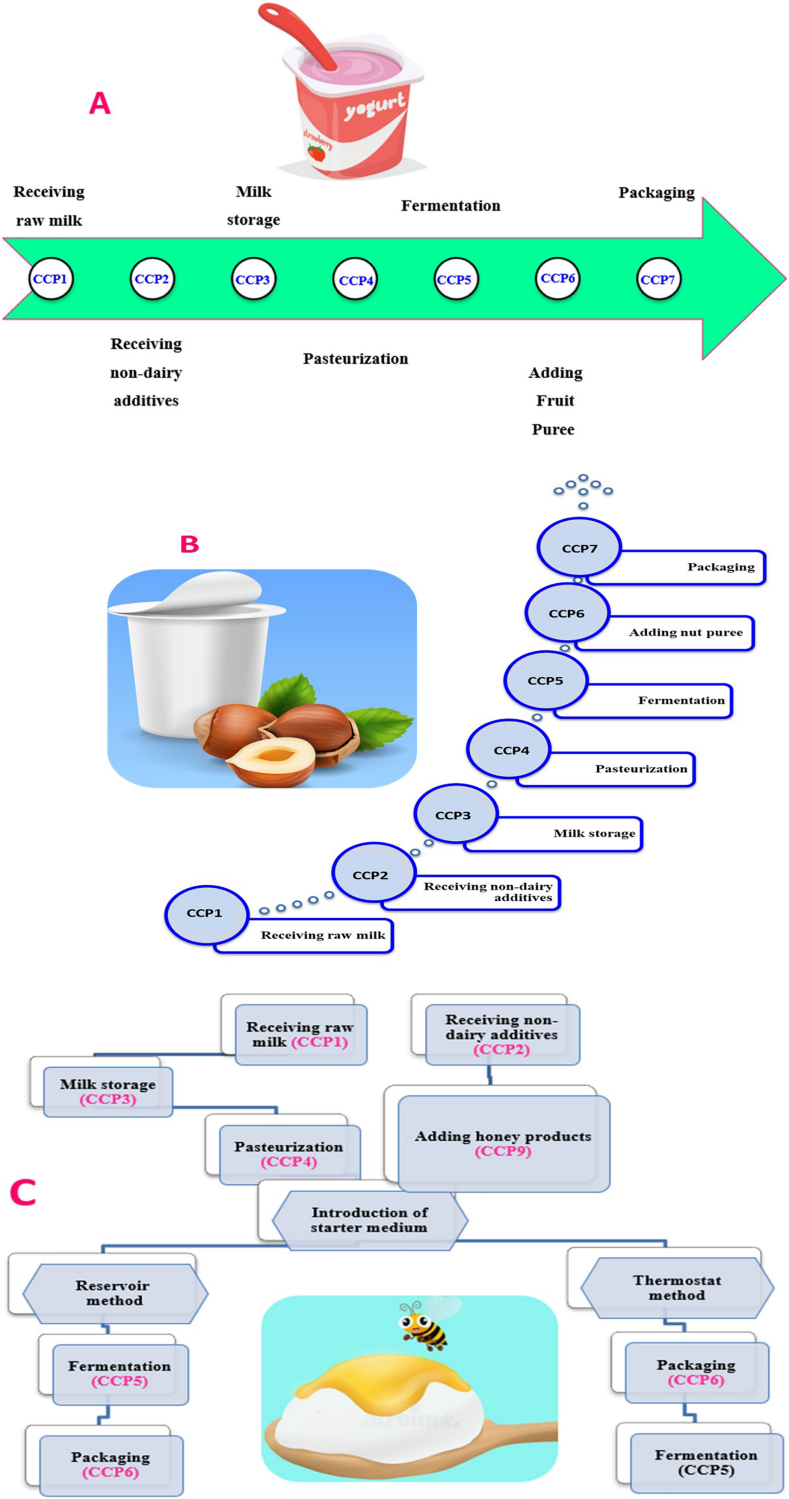
The main critical control points in manufacturing of yogurt with fruits (A), yogurt with nut puree (B), and yogurt with honey (C).

In addition to the previous six CCPs (Fig. 4A and Table 5), adding fresh fruits as in case of stirred yogurt with fruit puree should be considered as CCP number 7. The fresh fruits can be added post pasteurization due to their low pH nature which does not promote bacterial growth even that of *Clostridium botulinum* (Sandrou & Arvanitoyannis, 2000). In contrast, the addition of nut puree during the manufacture of yogurt ought to be considered as the CCP number 7, alongside with the previous six CCPs of plain set yogurt (Fig. 4B and Table 5). Adding nut in yogurt can be a source of microorganisms and toxins such as aflatoxin which exists in nuts if stored under moisture due to unsuitable environment of storage and harvesting (Emadi et al., 2021; Sandrou & Arvanitoyannis, 2000). There is great concern for nuts as one of the main aflatoxin-contaminated foods in human diet. The contamination of nuts with aflatoxins can be initiated in the field and during the different production processes including pre-harvest, harvest, post-harvest, storage, handling, and distribution (Abdolshahi et al., 2016). In this regard, implementation of good agricultural practice (GAP) and good manufacturing practice (GMP) will have a practical impact on the prevention of *Aspergillus* infection and aflatoxins formation at any point of nuts production stages (Campbell, Molyneux & Schatzki, 2003).

Production of yogurt with honey differs in processing than plain one. Honey is also added post pasteurization, due to its very low water activity as well as to sustain the prebiotic nature of honey (Merzlov et al., 2018). Likewise, adding honey represents the key CCP aside from the previous CCPs of plain set yogurt (Fig. 4C and Table 5).

## Conclusions and future recommendations

HACCP is an internationally implemented control system to ensure the safety of food products, especially in the fermented dairy industry. Its implementation is crucial for preventing food hazards that impose serious health risks on consumers and economic waste. Analysis of all types of hazards (biological, chemical, and physical) have been compiled for the raw materials, processing steps, and packaging typically employed to produce the different types of yogurts including the set and stirred ones. The decision tree application showed six critical control points (CCPs) in the production of plain set yogurt including: (1) receiving raw milk, (2) receiving non-dairy materials, (3) milk storage, (4) pasteurization, (5) fermentation, and (6) packaging. More CCPs are included in the production of stirred yogurt with fruit puree, nut puree, or honey, depending on the property and/or nature of each additive. Finally, preventive limits and control actions are presented to prevent or reduce potential hazards in each CCP, along with appropriate records, monitoring and verification where applicable. The HACCP system in dairy industry showed a great efficacy in eliminating serious milk-borne illnesses such as *Salmonella* as well as to prevent losses to the yogurt manufacturing facility itself. The findings of microbiological analysis of packed yogurt indicated that implementation of HACCP could enhance the microbial quality of yogurt. The implementation of the HACCP plan in a small-scale yogurt pilot plant has also brought benefits to food chains, especially saving foods against spoilage as more typical in developing countries. This system allows for immediate action when safety issues are identified, from the receipt of raw milk to the delivery of yogurt product, and it serves as the foundation for educational tools for practicing and learning the implementation of a food safety management system. The study’s main conclusions underlie future research regarding the expansion of food safety management systems by utilizing predictive microbiology models and risk-assessment schemes as an integrated model of good practice and education tools for quality control workers in the field of dairy products.

## Additional Information and Declarations

### Data Availability

The following information was supplied regarding data availability: This is a review and no raw data available.
